# Reference models for individualized assessment of cardiorespiratory fitness in children and adolescents with congenital heart disease: a retrospective multicentre study

**DOI:** 10.1007/s00431-025-06270-x

**Published:** 2025-06-26

**Authors:** Vibeke Klungerbo, Asle Hirth, Per Morten Fredriksen, René Holst, Elisabeth Edvardsen, Henrik Holmstrøm, Thomas Möller

**Affiliations:** 1 Department of Paediatric Cardiology, Oslo University Hospital, P.O. Box 4950, 0424 Nydalen, Oslo, Norway; 2 Institute of Clinical Medicine, Faculty of Medicine, University of Oslo, Oslo, Norway; 3 Department of Paediatrics, Haukeland University Hospital, Bergen, Norway; 4 Faculty of Applied Ecology, Agricultural Sciences and Biotechnology, University of Inland Norway, Hamar, Norway; 5 Faculty of Health, Welfare and Organization, Østfold University College, Fredrikstad, Norway; 6 Department of Biostatistics, Institute of Basic Medical Sciences, University of Oslo, Oslo, Norway; 7 Department of Pulmonary Medicine, Oslo University Hospital, Oslo, Norway

**Keywords:** Cardiopulmonary Exercise Test, Treadmill, Reference values, Congenital heart disease

## Abstract

Cardiopulmonary Exercise Testing (CPET) is considered the gold standard assessment of peak oxygen uptake ($\dot VO_2$peak), and consequently, cardiorespiratory fitness (CRF). Common practice in children with congenital heart diseases (CHD) is to compare individual test results to reference values in healthy children. This approach does not account for the diversity of heart defects and may also be demotivating for children with CHD. This retrospective multicentre study aimed to establish individualized CRF reference models specific to children with CHD, to facilitate a better understanding and management of their condition. A total of 1475 CPETs until exhaustion were performed in 943 children and adolescents at two hospitals in Norway. The children and adolescents were categorized into three groups: simple defects (VSD, ASD, CoA, LVOTO; *n* = 497, 38% female), moderate defects (TGA, Fallot; *n* = 299, 41% female), and univentricular defects with a Fontan circulation (*n* = 147, 42% female). Multivariable mixed-effects models with individuals as random intercepts were used to develop group-specific reference models for CRF. Estimated predictive models including covariance matrices for each group and outcome are provided, allowing for the generation of reference values with confidence intervals for clinical and research applications. *Conclusion:* Treadmill-based reference models for maximal and submaximal CRF variables in the most prevalent types of CHD are presented. Using individualized reference values has the potential to improve clinical decision-making for children with CHD and to enhance their sense of mastery. A web-based calculator is available for quick and easy clinical use.

**Graphical abstract:**

Graphical abstract: Created in BioRender. Klungerbo, V. (2025) https://BioRender.com/w93q182.

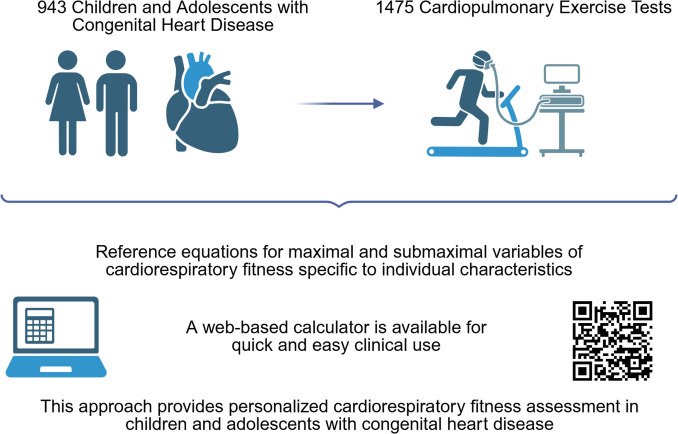

**Supplementary Information:**

The online version contains supplementary material available at 10.1007/s00431-025-06270-x.

**Table:** 

**What is Known:** • *Children with congenital heart disease (CHD) generally have reduced cardiorespiratory fitness (CRF) compared to healthy peers. Treadmill-based reference values for CRF in CHD are limited.*	
**What is New:** • *This study provides individualized, treadmill-based reference models for maximal and submaximal CRF variables in children with various CHD.* • *A web-based calculator is available for quick and easy clinical use.* • *This approach may improve the interpretation of CRF in children with CHD.*	

## Introduction

Congenital heart disease (CHD) with structural malformations is present in approximately 1% of all newborns, making it the most common birth defect [1, 2]. Today, most CHD cases can be repaired or palliated through surgical and catheter interventional methods, allowing for an almost normal lifespan [3], though many require lifelong follow-up and repeated interventions. Children and adolescents with CHD often have impaired cardiorespiratory fitness (CRF) due to various reasons, including unfavourable haemodynamics, impaired myocardial function, cyanosis, heart rhythm disturbances, residual defects [4], or habitually reduced physical activity level [5]. CRF impairment affects daily life activities and quality of life and is a major criterion for reinterventions. Furthermore, CRF is an important prognostic marker for morbidity and mortality in CHD [6, 7]. Thus, serial assessment of CRF by a cardiopulmonary exercise test (CPET) is an integrated and essential part of long-term follow-up in CHD children and adolescents [8].

A CPET is commonly conducted repeatedly during routine clinical evaluations with individual time intervals. Measurement of peak oxygen uptake ($\dot VO_2$peak) via CPET represents the gold standard assessment of CRF [9, 10]. This method is non-invasive and available in most tertiary centres. Reference values for CRF are influenced by individual variables such as sex, age, ethnicity, cultural factors, exercise habits, and diagnosis, as well as institutional factors like the choice of ergometer and protocols [11].

CRF assessments in children and adolescents are commonly compared to age- and gender-based normative data from the healthy population [12, 13]. However, in the paediatric CHD population, treadmill-based CRF reference values including various CHD, variables other than $\dot VO_2$peak, and generated and presented with sufficient methodological quality are lacking. Although studies that described CRF for the paediatric CHD population potentially may be used as reference values [12, 14–16], they have notable limitations. Some used cycle ergometry [14, 15], which cannot directly be compared to CPETs performed on a treadmill [17]. Some studies are limited by small sample sizes, heterogeneous populations, or insufficient methodology and outcome variables [12, 14–16]. There is a knowledge gap regarding robust, diagnosis-related, treadmill-based CRF reference values specifically tailored for children and adolescents with CHD. This limits the clinicians’ ability to interpret CRF accurately in this population.

This retrospective multicentre study aimed to establish CRF reference models assessed on a treadmill in Norwegian children and adolescents with CHD. The models should provide individualized predictive reference values of CRF.

## Materials and methods

### Study design and patient population

In this retrospective multicentre study, CPETs performed during 1996 and 2020 in children and adolescents with preselected diagnoses at Oslo University Hospital (OUH) and Haukeland University Hospital (HUH) were included. The study was conducted as a quality assurance project, as described in the ethics section. From 2005, the only surgical centre for children with CHD in Norway is OUH. All tests were conducted as part of routine clinical outpatient visits or hospital admissions. Inclusion criteria were children and adolescents between 6 and 18 years old diagnosed with isolated atrial or ventricular septal defects (ASD and VSD respectively), coarctation of the aorta (CoA), left ventricular outflow tract obstruction (LVOTO), tetralogy of Fallot (ToF), D-transposition of the great arteries repaired with an arterial switch operation (TGA), and palliated univentricular heart defects with Fontan circulation (Fontan) (Online resource 1). To achieve substantial group size for analysis, children and adolescents were categorized into three groups based on expected CPET performance [12, 18, 19]:

- Simple defects: ASD, VSD, COA, and LVOTO
- Moderate defects: ToF and TGA
- Univentricular defects with a Fontan circulation

Given CHD’s anatomical and functional diversity, defining large homogenous groups is challenging. Variations in defect size, location, presence of additional heart defects, and surgical/interventional procedures affect CRF. Clear inclusion criteria improve comparability and clinical utility, though it demands to extrapolate results in borderline cases or children and adolescents with combined defects. Other diagnoses were excluded due to clinical and hemodynamic heterogeneity that would make grading of disease severity challenging, in addition to significant additional cardiac abnormalities or conditions affecting CPET performance, such as neurocognitive or locomotive disorders, or major complications of CHD (e.g., Eisenmenger syndrome). Children and adolescents with pacemakers, implantable cardioverter-defibrillators, or beta blockers were also excluded. Submaximal CPETs based on a respiratory exchange ratio (RER) ≤ 1.00 were excluded, in alignment with previously published paediatric CRF studies [15, 20].

### Cardiopulmonary exercise test

Under guidance of an experienced exercise physiologist or physiotherapist and a physician, children and adolescents performed a maximal symptom-limited CPET on a treadmill (Technogym, Italy; Woodway, USA) using a stepwise Oslo protocol [21] or a modified ramped Bruce protocol until exhaustion [22]. Hand support was not permitted. Gas exchange and ventilatory variables were continuously determined sampling through a breathing mask and analysed breath-by-breath. Heart rate (HR) was recorded using a 12-lead ECG. $\dot VO_2$peak in absolute values (mL^.^min^−1^) and relative to body mass (mL^.^kg^−1^ min^−1^), in addition to HR, RER, minute ventilation (V_E_), ventilatory efficiency (V_E_/VCO_2_ slope), oxygen pulse (O_2_-pulse), and breathing frequency (BF) were registered. Three types of gas analysers were used at the two test centres: Oxycon Pro (Erich Jaeger GmBh), Sensor Medics Vmax 29 (CareFusion Corporation), and Vyntus CPX Metabolic Cart (CareFusion Corporation, Höchberg, Germany). All analysers were gas and volume calibrated before each test according to manufacturer’s standards.

### Definitions of variables

Gas exchange variables and V_E_ were averaged over 30-s intervals. The O_2_-pulse was calculated by dividing $\dot VO_2$peak (mL) by HRpeak from the same 30-s time interval as $\dot VO_2$peak. The *V*_E_/VCO_2_ slope was calculated based on *V*_E_ and carbon dioxide (CO_2_) responses during exercise, where values after the respiratory compensation point were excluded [9]. Body mass index (BMI) was calculated as body weight (kg) divided by height squared in metres (kg/m^2^).

### Statistical analysis

Descriptive results are presented as means and standard deviation (SD) or as counts with percentages (%).

To account for correlation in repeated CPETs within children and adolescents, a mixed-effects regression analysis was used, with individuals as random intercepts. Multicollinearity between covariates were examined by means of exploratory graphs and calculation of variance inflation factors. This revealed a strong association between age and height, and between weight and BMI. Consequently, only height and BMI were included, as height was considered a better predictor in clinical settings, and both were stronger predictors statistically. To assess for potential residual age effects, we examined model residuals plotted against age and found no clear pattern, suggesting that height adequately captured age-related variation.

Normality of continuous variables and model residuals was assessed using Q-Q plots and residual plots. For non-normal data, a Box-Cox analysis was conducted to identify optimal power transformations to achieve normality (Online resource 2). In these cases, estimations were performed on the transformed scale, followed by back transformation for obtaining estimates on the relevant clinical scales.

Currently, only the Vyntus CPX Metabolic Cart is used in our hospitals; therefore, data from the older software versions, Oxycon Pro and Sensor Medics Vmax, were combined and analysed against the Vyntus CPX software. The initial model included sex, height, BMI, software, and hospital as explanatory variables. Additionally, interaction terms between sex and height, and between sex and BMI, allowed for sex-specific effects on the outcomes. The full models were reduced through backward elimination to identify all significant variables for each group. At each step, the term with the highest *P* value above 0.05 was removed, followed by a re-estimation. This process was repeated until all explanatory variables reached significance, with a few exceptions in cases with borderline significance. The intraclass correlation (ICC) was used to explore the effects of individual variability in the models (Online resource 3).

Missing data for the *V*_E_/VCO_2_ slope (16%) were addressed using the multiply imputation method. Analyses indicated that missing values for BF did not exceed 5% (3.6% missing); therefore, nothing was done to handle these.

To establish reference intervals, covariance matrices with 95% confidence intervals (CIs) were used. Unfortunately, this was not possible for the outcome *V*_E_/VCO_2_ slope due to the use of multiply imputation. The final mixed-effects models are presented in Online resource 3. To ensure reproducibility, the associated covariance matrices are presented in Online resource 4. The significance level was set at 0.05, and the CI at a standard 95%. All analyses were performed using Stata version 17.0 (StataCorp, College Station, Texas, USA).

## Results

### Study population

The database contained 1475 tests from 943 individual children diagnosed with ASD, VSD, CoA, LVOTO, ToF, TGA, and univentricular defects with a Fontan circulation. Background information is provided in Table 1.

**Table 1:** Characteristics of the children and adolescents, stratified for group and diagnosis

	***n***	Male (%)	Age	Height	Weight	BMI	$\dot VO_2$peak (mL^.^min^−1^)	$\dot VO_2$peak (mL^.^kg^−1^ min^−1^)	HRpeak	VEpeak	RER	BF	O_2_-pulse	***V***_E_/VCO_2_
**Simple**	**701**	**441 (63%)**	**13.3 ± 2.9**	**157.3 ± 16.8**	**49.6 ± 16.9**	**19.4 ± 3.7**	**2182.5 ± 805.1**	**44.6 ± 8.9**	**191.0 ± 11.6**	**76.5 ± 27.0**	**1.12 ± 0.09**	**54.2 ± 11.2**	**11.4 ± 4.1**	**28.2 ± 5.0**
ASD	45	17 (38%)	13.7 ± 3.0	155.1 ± 15.6	48.5 ± 14.0	19.7 ± 3.0	2055.7 ± 696.3	43.1 ± 9.8	192.2 ± 11.0	74.0 ± 21.3	1.11 ± 0.07	55.3 ± 13.0^a^	10.7 ± 3.7	30.0 ± 6.1^b^
VSD	146	82 (56%)	13.1 ± 2.9	157.1 ± 17.3	48.6 ± 16.8	19.0 ± 3.6	2196.5 ± 833.1	45.7 ± 9.1	191.4 ± 13.0	75.7 ± 26.3	1.11 ± 0.08	54.2 ± 11.1^c^	11.4 ± 4.2	28.0 ± 5.1^d^
CoA	231	137 (59%)	13.3 ± 3.1	156.8 ± 17.2	49.6 ± 17.5	19.5 ± 3.7	2131.6 ± 803.3	43.5 ± 8.8	189.8 ± 10.7	74.7 ± 26.5	1.13 ± 0.10	54.9 ± 11.7^e^	11.2 ± 4.2	28.0 ± 5.0^f^
LVOTO	279	205 (74%)	13.2 ± 2.8	158.1 ± 16.4	50.3 ± 17.0	19.5 ± 3.9	2237.7 ± 807.1	45.1 ± 8.9	191.6 ± 11.6	78.8 ± 28.7	1.11 ± 0.09	53.5 ± 10.7^ g^	11.7 ± 4.1	28.3 ± 4.9^ h^
**Moderate**	**497**	**281 (57%)**	**13.1 ± 3.1**	**155.3 ± 16.9**	**47.4 ± 16.6**	**19.0 ± 3.6**	**1876.6 ± 687.7**	**40.6 ± 8.8**	**187.6 ± 12.5**	**70.4 ± 25.5**	**1.11 ± 0.08**	**54.1 ± 10.7**	**10.0 ± 3.6**	**29.8 ± 5.6**
Fallot	303	163 (54%)	13.3 ± 3.1	156.2 ± 16.6	47.7 ± 16.4	18.9 ± 3.6	1852.8 ± 675.9	39.8 ± 8.7	186.8 ± 13.7	69.1 ± 25.4	1.11 ± 0.08	53.8 ± 10.3^i^	9.9 ± 3.5	29.7 ± 5.5^j^
TGA	194	118 (61%)	12.7 ± 3.1	154.0 ± 17.2	47.0 ± 17.0	19.1 ± 3.5	1913.7 ± 706.0	41.8 ± 8.9	188.7 ± 10.2	74.5 ± 25.5	1.12 ± 0.09	54.4 ± 11.2^ k^	10.1 ± 3.7	30.1 ± 5.8^ l^
**Fontan**	**277**	**169 (61%)**	**13.1 ± 3.0**	**153.5 ± 16.9**	**45.3 ± 14.5**	**18.7 ± 3.0**	**1500.8 ± 561.8**	**33.6 ± 7.8**	**176.4 ± 17.3**	**65.6 ± 23.9**	**1.09 ± 0.07**	**53.4 ± 10.5**^**m**^	**8.6 ± 3.2**	**35.9 ± 6.0**^**n**^

Data are presented as counts (%) or means ± standard deviations

*ASD*; atrial septal defect, *VSD*; ventricular septal defects, *CoA*; coarctation of the aorta, *LVOTO*; left ventricular outflow tract obstruction, *Fallot*; tetralogy of Fallot, *TGA*; transposition of the great arteries, *Fontan*; univentricular defects with a Fontan circulation

^a^10 missing values, ^b^14 missing values, ^c^17 missing values,^d^29 missing values, ^e^5 missing values, ^f^36 missing values,^g^4 missing values, ^h^46 missing values, ^i^8 missing values, ^j^40 missing values, ^k^6 missing values, ^l^42 missing values, ^m^3 missing values, ^n^32 missing values

### Explanatory example for factor analysis and model building ($\dot VO_2$peak in Fontan circulation)

To obtain the most precise predictive models, separate analyses were conducted for each outcome within each of the three groups, resulting in a total of 21 sequential analyses. To facilitate quick interpretation and calculation of predicted values and corresponding confidence intervals, we developed a web-based app (ocbe.shinyapps.io/kids-chd/) [23]. The children and adolescents’ sex, anthropometric values, CHD group, and measured values are entered into the calculator. For ease of presentation, we provide example calculations for a single outcome. Similar calculations apply to all outcomes.

Box-Cox analysis revealed no need for transformation of the outcome or explanatory variables for weight-indexed $\dot VO_2$peak for children and adolescents with a Fontan circulation. Seven explanatory variables were initially included in the mixed-effects model. Only BMI, software, and hospital remained significant, along with the interaction between sex and height (Table 2), which implies no effect of height for females but an increasing effect for males.

**Table 2:** Mixed-effects model for $\dot VO_2$peak mL^.^kg^−1^ min^−1^ for Fontan children and adolescents, limited to three decimals. Note: a dummy variable male has the value 1 if the individual is male, otherwise 0

$\dot VO_2$peak mL^.^kg^*−*1^ min^*−*1^					
Effect	Estimate	Std. error	***P*** value	95% CI	
*β*_BMI_	*− *0.668	0.135	< 0.001	*− *0.933	*− *0.404
*β*_height-male_	0.033	0.007	< 0.001	0.020	0.046
*β*_software_ Vyntus CPX	* − *4.523	1.934	0.019	* − *8.314	* − *0.732
*β*_hospital_ Haukeland	3.746	1.244	0.003	1.308	6.183
Intercept	42.388	2.679	< 0.001	37.138	47.638

### Model equation

The *j*th observation for the *i*th individual is modelled by

$$\dot VO_2peak_{ij}\left(mL\cdot\;kg^{-1}min^{-1}\right)=\beta_0+\beta_{BMI}\cdot BMI_{ij}+\beta_{height\;x-male}\cdot height\;x-male_{ij}+\beta_{software}\cdot software_{ij}+\beta_{hospital}\cdot hospital_{ij}+b_i+\varepsilon_{ij,}$$

where $$b_i\sim N\left(0,\;\sigma_b^2\right)\;and\;\varepsilon_{ij}\sim N\left(0,\sigma_\varepsilon^2\right)$$ 

Here, ${\beta }_{0}$ is the intercept of the model. When all predictor variables are zero, this represents the mean baseline value of $\dot VO_2$peak, in this case 42.39 mL^.^kg^−1^ min^−1^ (Table 2). The other $\beta$ coefficients are the effect sizes for the predictors. Specifically, ${\beta }_{BMI}$ represents the effect size of BMI, where an increase of 1 unit in BMI corresponds to a decrease of 0.67 mL^.^kg^−1^ min^−1^ in $\dot VO_2$peak. The ‘height x-male’ term represents the effect of height for males, indicating that for every additional centimetre in height for a male, there is an increase of 0.03 mL^.^kg^−1^ min^−1^, whereas there is no effect for females. The ‘software’ term represents the effect of software, with a decrease of 4.52 mL^.^kg^−1^ min^−1^ when using the Vyntus CPX software as opposed to using Oxycon Pro/Sensor Medics Vmax. Correspondingly, ‘hospital’ accounts for the estimated difference in $\dot VO_2$peak between hospitals caused by lab facilities, higher frequency of more severe CHD in case of pre-intervention CPET in Oslo, individual test supervisor performance, etc. If the individual is assessed at Haukeland rather than Oslo, there is an increase of 3.75 mL^.^kg^−1^ min^−1^. The term ${b}_{i}$ represents the random deviation of the *i*th individual from the overall mean, while $\varepsilon_{ij}$ represents the deviation of the *j*th observation within the mean of the *i*th individual. For some outcomes, regression is performed on a transformed scale, and the result is obtained by back-transformation.

### Inserting the estimated effects, the formula becomes

$$\dot VO_2\mathrm{peak}\;\left(\mathrm{mL}\cdot\;\mathrm{kg}^{-1}\;\min\nolimits^{-1}\right)=42.39-0.67\cdot\mathrm{BMI}+0.03\cdot\mathrm{height}\cdot1_{\left\{sex=male\right\}}-4.52\cdot1_{\left\{\mathrm{software}=\mathrm{Vyntus}\;\mathrm{CPX}\right\}}+3.75\cdot1_{\left\{\mathrm{hospital}=\mathrm{Haukeland}\right\}\cdot}$$

Here ${1}_{\left\{sex=male\right\}}$ is an indicator variable, taking the value 1 if the data point concerns a male and 0 if it concerns a female, and similarly for the other indicator variables.

By way of example consider a female individual with a BMI of 19, a height of 110 cm measured with the Oxycon Pro software at Oslo University Hospital. The predicted value of $\dot VO_2$peak for this individual is thus given by

$$\dot VO_2\mathrm{peak}\;\left(\mathrm{mL}\cdot\;\mathrm{kg}^{-1}\min\nolimits^{-1}\right)=42.39-0.67\cdot19+0.03\cdot110\cdot0-4.52\cdot0+3.75\cdot0=29.66.$$

For a male, with similar measures of BMI and height, measured with the Vyntus CPX software at Haukeland, the predicted $\dot VO_2$peak becomes

$$\dot VO_2\mathrm{peak}\;\left(\mathrm{mL}\cdot\;\mathrm{kg}^{-1}\min\nolimits^{-1}\right)=42.39-0.67\cdot19+0.03\cdot110\cdot1-4.52\cdot1+3.75\cdot1=32.19.$$

A significant effect of hospital and software was found; however, predictions should be applicable irrespective of the origin of the hospital or software. Therefore, for application, only BMI and height were considered, whereas software and hospital are omitted and integrated out of the equation using a weighted average (Table 1 in Online resource 5), with weights given by the proportion of observations taken at each combination. For example, for a male Fontan child or adolescent with a BMI of 21 and height of 160 cm examined with the Oxycon Pro/Sensor Medics Vmax at Haukeland, the predicted $\dot VO_2$peak would be 37.38 mL^.^kg^−1^ min^−1^ (configuration 1 in Online resource 5). After weighted average for hospital and software, the predicted $\dot VO_2$peak is 34.20 mL^.^kg^−1^ min^−1^ with a corresponding 95% CI of 32.72 to 35.69 mL^.^kg^−1^ min^−1^. The intraclass correlation revealed that 65% of the variance was *between* individuals, with the remaining 35% attributable to *within* individual variation (Table 26 in Online resource 3).

## Discussion

Reference models for CRF variables assessed on a treadmill in children and adolescents with CHD are presented. Mixed-effects models were estimated for several outcomes in each CHD group. This allows for child or adolescent-specific reference values rather than groupwise averages. A web-based calculator is available for quick and easy individual prediction in clinical settings.

Sex significantly influenced most CPET variables, primarily through interaction with anthropometric variables. A sex-dependent effect of BMI was identified only in Fontan children and adolescents, more specifically in absolute $\dot VO_2$peak, HRpeak, and O_2_-pulse. Predictors for absolute $\dot VO_2$peak, *V*_E_, and O_2_-pulse were consistent across simple and moderate defects.

Our methodological approach to presenting CPET reference data marks a departure from the traditional use of group averages and distribution-based metrics, primarily *Z*-scores. While this conventional method is intuitive and widely applied, it has important limitations, particularly in accurately assessing individual results and their clinical relevance for each patient. Our reference models aim to overcome these shortcomings through individualized predictive calculations, making averages and *Z*-scores obsolete. These models also can significantly enhance CPET interpretation by allowing comparisons to a CHD-specific population, in addition to tracking individual CRF trajectories [24]. They may also improve CPET experience for children and their caregivers by avoiding the inevitable negative message of major deviations from ‘normal’ results even after a well-performed test [25]. Further, this may foster mastery and motivation to engage in physical activity. This is crucial, as childhood is a critical period for establishing regular physical activity and healthy behaviours, which contribute to fitness and thereby offer long-term benefits [5, 26].

### General considerations and pitfalls for CPET reference values

Determining reference values for CPET in paediatric populations is challenging due to growth and maturation effects on CRF [11]. Puberty introduces sex-specific changes at varying ages, including increased muscle mass, higher oxygen-carrying capacity, and larger heart size in males [27]. Current body size adjustments are insufficient [11, 28]. CHD children and adolescents with severe defects may experience slow growth and delayed maturation [29, 30], particularly in Fontan circulation, affecting height and BMI [31, 32]. Thus, height has been used as a CRF metric instead of age [15]. While percent fat-free mass obtained from dual-energy x-ray absorptiometry (DEXA) might better scale CRF than body weight or BMI [33], it is impractical in clinical settings and not widely in use. Therefore, adjustments for multiple body size parameters are necessary and BMI was also included in our models, despite its limitations, as it does not differentiate between fat and lean mass. This adds to the existing bias from the conventional approach of indexing $\dot VO_2$peak to body weight [34], though widely used in paediatric populations. Using a correction factor, such as an exponential factor of 0.67 has been discussed to reduce the effect of body mass [34], as increasing body weight during adolescence may obscure increases in $\dot VO_2$peak. However, due to the low clinical utilization of the 0.67 correction, we used an uncorrected weight adjustment. Thus, in clinical settings, having reference values for both absolute and relative measures is crucial for accurately assessing individual CRF. Ultimately, tracking individual development using consistent reference values over repeated tests is essential, as illustrated in a case report [35].

### Methodological discussion

Previous studies on CRF in paediatric CHD have often focused on a single CRF variable or structural defect. A multicentre retrospective Dutch study included a robust sample of various CHD assessed on a cycle ergometer, but did not account for the correlation induced by repeated measurements in their statistics [15]. Ignoring this will underestimate the standard errors and thereby not provide reliable estimates of the uncertainty. Our findings indicate that 35% of the variation in V̇O_2_peak (ml^.^kg^−1^ min^−1^) in Fontan children and adolescents is attributable to within-individual variation, underscoring the importance of using statistical methods that account for repeated measures. The Dutch study standardized CPET variables to height and stratified for sex. Our approach offers a more comprehensive and precise analysis and prediction, accounting for both fixed and random variability, providing robust insights into each factor’s contribution while ensuring reliable and generalizable findings. However, models with a high level of within-individual variation imply reduced sensitivity in detecting small changes. This is particularly relevant in Fontan children and adolescents, where clinical interpretation remains essential when evaluating marginal changes over time.

A cross-sectional cycle ergometer study from France included 496 children with various CHD but only reported $\dot VO_2$peak [14]. Treadmill is more demanding than cycling, resulting in higher $\dot VO_2$peak in both healthy children [36] and those with CHD [37]. Thus, reference values obtained from cycle ergometry cannot be directly applied to CPETs performed on a treadmill. An important but often overlooked factor is the variability between metabolic carts, which may contribute to differences in $\dot VO_2$peak and other measurements from CPET [38], potentially diminishing the observed difference between ergometers. However, by including software in the models, we accounted for these potential discrepancies.

### Cultural variations

Cultural and geographic differences can significantly influence $\dot VO_2$peak, particularly due to differences in habitual physical activity levels. A cross-sectional observational study from China evaluated 182 children with CHD on a treadmill [16]. Norwegian populations have been shown to exhibit high levels of physical fitness [13]. Therefore, comparisons with populations from other regions are complicated by cultural and lifestyle differences, including the frequency and type of physical activities children typically engage in. Considering this, in more sedentary or demographically different populations, such as those with lower physical activity levels, differing socioeconomic backgrounds, or ethnic diversity, the presented models may overestimate expected CRF. These factors underscore the importance of utilizing reference values that reflect the specific characteristics of the population being assessed. In this context, a prior Norwegian study assessed 187 children with CHD using a treadmill [12]. While this study focused on a similar population and geographic context, it included few CRF variables [12], highlighting the need for region-specific data to accurately interpret $\dot VO_2$peak and other CRF-related measures.

### Calculator and recommendations for clinical interpretation

The calculator (ocbe.shinyapps.io/kids-chd/) allows clinicians to interpret CRF in relation to individualized normative data. This can help prevent discouragement and foster motivation for physical activity in children and adolescents with CHD. While the tool facilitates rapid CRF comparison to individually predicted values, its clinical utility relies on appropriate interpretation in context. We suggest that clinicians use the models primarily to track changes over time, rather than relying on a single measurement for comparison. In patients with more complex defects or signs of deterioration, more frequent testing may be considered, guided by clinical judgment. However, we do not propose strict thresholds for concern, as these may vary depending on diagnosis, functional status, and individual trajectory. While aiding decision-making, the calculator should complement serial testing and thorough clinical evaluation of the child or adolescent for optimal management.

### Strengths and limitations

This project benefits from a large sample size and the use of a treadmill, representing a methodological advancement over previous paediatric CHD research. To account for correlation from repeated measurements, mixed-effects models were employed. Including multiple independent variables in the regression analysis enabled the development of robust predictive models. Differences between hospitals and software were addressed by including these as fixed effects and integrating them out through weighted averages.

Limitations include the retrospective design. CPETs were performed at two tertiary CHD centres, which may introduce selection bias by including children and adolescents with more reduced CRF. Due to the retrospective design, certain information (e.g., individual functional variations, pubertal status, and physical activity levels) was unavailable. Thus, unmeasured maturational differences may still influence individual CRF levels. These factors should be considered in future studies to improve predictive precision. We did not differentiate between left or right ventricular dominance in children and adolescents with univentricular circulation. The dataset spans from 1996 to 2020, which raises potential concerns about temporal changes in CHD treatment, fitness trends, and equipment. However, including older data was necessary to achieve sufficient sample size and is consistent with approaches used in other retrospective reference studies. Because the dataset includes both routine outpatient and symptom-driven tests, this may slightly lower predicted values and should be considered in clinical interpretation. We did not include VO_2_ at the ventilatory anaerobic threshold due to inconsistent determination, which limited its reliability. The grouping of diagnoses into broader categories may overlook diagnosis-specific differences in CRF; this heterogeneity could influence model precision and should be considered when interpreting results. Finally, not all children and adolescents had complete data sets due to technical issues.

## Conclusions

Treadmill-based reference models for maximal and submaximal CRF variables in the most prevalent types of CHD are presented. Using individualized reference values, providing individual CRF trajectories, might improve the clinical decision-making of children with CHD.

## Supplementary Information

Below is the link to the electronic supplementary material.

Supplementary file1 (PDF 154 kb)

Supplementary file2 (PDF 200 kb)

Supplementary file3 (PDF 467 kb)

Supplementary file4 (PDF 470 kb)

Supplementary file5 (PDF 273 kb)

Supplementary file6 (PDF 230 kb)

## Data Availability

The data underlying this article will be shared on reasonable request to the corresponding author.

## Abbreviations

ASD: Atrial septal defect
BF: Breathing frequency
BMI: Body mass index
CHD: Congenital heart disease
CI: Confidence interval
CO_2_: Carbon dioxide
CoA: Coarctation of the aorta
CPET: Cardiopulmonary exercise test
CRF: Cardiorespiratory fitness
DEXA: Dual-energy x-ray absorptiometry
HR: Heart rate
HUH: Haukeland University Hospital
ICC: Intraclass correlation
LVOTO: Left ventricular outflow tract obstruction
O_2_-pulse: Oxygen pulse
OUH: Oslo university hospital
RER: Respiratory exchange ratio
SD: Standard deviation
TGA: Transposition of the great arteries
ToF: Tetralogy of Fallot
V_E_: Minute ventilation
V_E_/VCO_2_ slope: Ventilatory efficiency
V̇O_2_peak: Peak oxygen uptake
VSD: Ventricular septal defect
