# Supplementary material for: Reference models for individualized assessment of cardiorespiratory fitness in children and adolescents with congenital heart disease: a retrospective multicentre study
Source: Eur J Pediatr. 2025 Jun 26;184(7):450. doi: 10.1007/s00431-025-06270-x (PMC12202686; doi:10.1007/s00431-025-06270-x)
Supplement: Supplementary file 1 — (PDF 154 kb) [file 431_2025_6270_MOESM1_ESM.pdf]

Online supplement 1

Reference models for individualized assessment of cardiorespiratory fitness in children and adolescents with congenital heart disease: a retrospective multicentre study

European Journal of Pediatrics

Vibeke Klungerbo[a,b], Asle Hirth[c], Per Morten Fredriksen[d,e], René Holst[f], Elisabeth Edvardsen[g], Henrik Holmstrøm[b], Thomas Möller[a]

a) Department of Paediatric Cardiology, Oslo University Hospital, Oslo, Norway
b) Institute of Clinical Medicine, Faculty of Medicine, University of Oslo, Oslo, Norway
c) Department of Paediatrics, Haukeland University Hospital, Bergen, Norway
d) Faculty of Applied Ecology, Agricultural Sciences and Biotechnology, University of Inland Norway, Hamar, Norway
e) Faculty of Health, Welfare and Organization, Østfold University College, Fredrikstad, Norway
f) Department of Biostatistics, Institute of Basic Medical Sciences, University of Oslo, Oslo, Norway
g) Department of Pulmonary Medicine, Oslo University Hospital, Oslo, Norway

Corresponding author:

Vibeke Klungerbo

Dept. of Paediatric Cardiology

Oslo University Hospital

P.O. Box 4950 Nydalen, 0424 Oslo, Norway

Phone: +47 23070000

Fax: +47 23072330

E-mail: vibklu@ous-hf.no

ORCID: 0000-0003-0980-0971

Supplement 1

<u>Inclusion criteria:</u>

ASD (closed): Atrial septum defects of secundum type or sinus venosus type, closed by surgical or interventional procedure.

VSD (closed + small open): Isolated ventricular septal defect without other major congenital cardiac malformations. The VSD should have been closed by surgical or interventional procedures. In the case of a minor open VSD considered below the closure threshold, there should be no sign of volume overload or pulmonary hypertension (Eisenmenger physiology). Concomitant ASD or right ventricular outflow tract obstruction is no exclusion criterion if the ASD has been closed and the residual RVOTO is of not more than mild degree.

Aortic stenosis: Left ventricular outflow tract obstruction at the subvalvular, valvular, or supravalvular level without other major congenital cardiac malformations and aortic arch pathology.

Coarctation: Stenosis of the aortic arch either as a hypoplastic arch or distinct coarctation. In any case, the obstruction should have been addressed by surgical or interventional procedure, or the lesion should be considered below the intervention threshold.

Fallot: Typical anatomy surgically corrected.

Transposition (arterial switch): Transposition of the great arteries surgically corrected by the arterial switch procedure (Jatene) and not corrected by a Mustard/Senning or Rastelli-type procedure.

Fontan/TCPC: Functional univentricular circulation of any ventricular morphology without a pumping chamber supporting the pulmonary circulation.
