# Supplementary material for: Reference models for individualized assessment of cardiorespiratory fitness in children and adolescents with congenital heart disease: a retrospective multicentre study
Source: Eur J Pediatr. 2025 Jun 26;184(7):450. doi: 10.1007/s00431-025-06270-x (PMC12202686; doi:10.1007/s00431-025-06270-x)
Supplement: Supplementary file 2 — (PDF 200 kb) [file 431_2025_6270_MOESM2_ESM.pdf]

Online supplement 2

Reference models for individualized assessment of cardiorespiratory fitness in children and adolescents with congenital heart disease: a retrospective multicentre study

European Journal of Pediatrics

Vibeke Klungerbo[a,b], Asle Hirth[c], Per Morten Fredriksen[d,e], René Holst[f], Elisabeth Edvardsen[g], Henrik Holmstrøm[b], Thomas Möller[a]

a) Department of Paediatric Cardiology, Oslo University Hospital, Oslo, Norway
b) Institute of Clinical Medicine, Faculty of Medicine, University of Oslo, Oslo, Norway
c) Department of Paediatrics, Haukeland University Hospital, Bergen, Norway
d) Faculty of Applied Ecology, Agricultural Sciences and Biotechnology, University of Inland Norway, Hamar, Norway
e) Faculty of Health, Welfare and Organization, Østfold University College, Fredrikstad, Norway
f) Department of Biostatistics, Institute of Basic Medical Sciences, University of Oslo, Oslo, Norway
g) Department of Pulmonary Medicine, Oslo University Hospital, Oslo, Norway

Corresponding author:

Vibeke Klungerbo

Dept. of Paediatric Cardiology

Oslo University Hospital

P.O. Box 4950 Nydalen, 0424 Oslo, Norway

Phone: +47 23070000

Fax: +47 23072330

E-mail: vibklu@ous-hf.no

ORCID: 0000-0003-0980-0971

Supplement 2

Transformations applied to outcomes and explanatory variables for each group

Table 1 Transformations of outcomes and explanatory variables. n/a: not applicable.

	Simple defects		Moderate defects		Univentricular defects with Fontan circulation	
	**Outcome**	**BMI**	**Outcome**	**BMI**	**Outcome**	**BMI**
**$\dot{V}O_2$peak mL·min^{-1}**	log	log	log	log	log	log
**$\dot{V}O_2$peak mL·kg^{-1}·min^{-1}**	n/a	n/a	n/a	log	n/a	n/a
**Heart rate**	^4.3	n/a	^5	n/a	^3.5	n/a
**Ventilation**	log	^-3.6	log	log	log	log
**Oxygen pulse**	log	^-1.7	log	log	log	n/a
**Ventilatory efficiency**	^-0.4	log	^-0.4	log	log	log
**Breathing frequency**	^0.4	log	^0.6	log	log	log
