# Supplementary material for: Reference models for individualized assessment of cardiorespiratory fitness in children and adolescents with congenital heart disease: a retrospective multicentre study
Source: Eur J Pediatr. 2025 Jun 26;184(7):450. doi: 10.1007/s00431-025-06270-x (PMC12202686; doi:10.1007/s00431-025-06270-x)
Supplement: Supplementary file 3 — (PDF 467 kb) [file 431_2025_6270_MOESM3_ESM.pdf]

Online supplement 3

Reference models for individualized assessment of cardiorespiratory fitness in children and adolescents with congenital heart disease: a retrospective multicentre study

European Journal of Pediatrics

Vibeke Klungerbo[a,b], Asle Hirth[c], Per Morten Fredriksen[d,e], René Holst[f], Elisabeth Edvardsen[g], Henrik Holmstrøm[b], Thomas Möller[a]

a) Department of Paediatric Cardiology, Oslo University Hospital, Oslo, Norway
b) Institute of Clinical Medicine, Faculty of Medicine, University of Oslo, Oslo, Norway
c) Department of Paediatrics, Haukeland University Hospital, Bergen, Norway
d) Faculty of Applied Ecology, Agricultural Sciences and Biotechnology, University of Inland Norway, Hamar, Norway
e) Faculty of Health, Welfare and Organization, Østfold University College, Fredrikstad, Norway
f) Department of Biostatistics, Institute of Basic Medical Sciences, University of Oslo, Oslo, Norway
g) Department of Pulmonary Medicine, Oslo University Hospital, Oslo, Norway

Corresponding author:

Vibeke Klungerbo

Dept. of Paediatric Cardiology

Oslo University Hospital

P.O. Box 4950 Nydalen, 0424 Oslo, Norway

Phone: +47 23070000

Fax: +47 23072330

E-mail: vibklu@ous-hf.no

ORCID: 0000-0003-0980-0971

Supplement 3

Simple defects

Weighted averages by hospital and software configurations

Table 1 Simple defects. Weighted average for configurations of hospital and software.

Configuration	Hospital	Software	Weighting
1	Haukeland	Oxycon Pro/Sensor Medics Vmax	36.52%
2	Haukeland	Vyntus CPX	4.99%
3	Oslo	Oxycon Pro/Sensor Medics Vmax	58.20%
4	Oslo	Vyntus CPX	0.29%

Table 2. Simple defects. Weighted average for hospital.

Configuration	Hospital	Weighting
1	Haukeland	41.51%
2	Oslo	58.49%

Table 3 Simple defects. Weighted average for software.

Configuration	Software	Weighting
1	Oxycon Pro/Sensor Medics Vmax	94.72%
2	Vyntus CPX	5.28%

Intraclass correlation coefficients

Table 4 Intraclass correlation coefficients (ICC), variance of random intercept, and residual variance for each outcome in the mixed-effects models for simple defects

Outcome	ICC	Variance of random intercept	Residual variance
**V̇O2peak mL·min^{-1}**	0.519	0.0133789	0.0124117
**V̇O2peak mL·kg^{-1}min^{-1}**	0.526	24.20802	21.85879
**Heart rate**	0.544	1.37e+18	1.15e+18
**Ventilation**	0.445	0.0172219	0.0215027
**Oxygen pulse**	0.506	0.0116404	0.0113449
**Ventilatory efficiency**	0.284	0.00006155	0.00015524
**Breathing frequency**	0.545	0.0691017	0.0577349

Mixed-effects model for $\dot{V}O_2$peak ($mL \cdot min^{-1}$)

Table 5 Simple defects. Note: a dummy variable male has the value 1 if the individual is male, otherwise 0.

**Log($\dot{V}O_2$ $mL \cdot min^{-1}$)**					
**Effect**	**Estimate**	**Std. Error**	**P value**	**95% CI**	
**Height**	0.0155584	0.0004598	<.001	0.0146573	0.0164596
**Log(BMI)**	0.4371531	0.0431555	<.001	0.3525699	0.5217363
**Height male**	0.0009139	0.0000923	<.001	0.000733	0.0010948
**Software Vyntus CPX**	-0.1803019	0.0268824	<.001	-0.2329905	-0.1276133
**Hospital Haukeland**	0.102317	0.0143184	<.001	0.0742535	0.1303805
**Intercept**	3.760053	0.0993233	<.001	3.565382	3.954723

Mixed-effects model for $\dot{V}O_2$peak ($mL \cdot kg^{-1} min^{-1}$)

Table 6 Simple defects. Note: a dummy variable male has the value 1 if the individual is male, otherwise 0.

**$\dot{V}O_2$ $mL \cdot kg^{-1} min^{-1}$**					
**Effect**	**Estimate**	**Std. Error**	**P value**	**95% CI**	
**Height**	0.0983195	0.0187146	<.001	0.0616395	0.1349995
**BMI**	-1.152879	0.0870345	<.001	-1.323463	-0.9822944
**Height male**	0.0423992	0.0038968	<.001	0.0347616	0.0500369
**Software Vyntus CPX**	-7.601633	1.13379	<.001	-9.823821	-5.379445
**Hospital Haukeland**	4.73933	0.605223	<.001	3.553115	5.925546
**Intercept**	45.77055	2.291847	<.001	41.27862	50.26249

Mixed-effects model for heart rate

Table 7 Simple defects.

**Heart rate$^{4.3}$**					
**Effect**	**Estimate**	**Std. Error**	**P value**	**95% CI**	
**Height**	9168804	3272220	0.005	2755371	1.56E+07
**Intercept**	5.13E+09	5.20E+08	<.001	4.11E+09	6.14E+09

Mixed-effects model for ventilation

Table 8 Simple defects. Note: a dummy variable male has the value 1 if the individual is male, otherwise 0.

**Log(Ventilation)**					
**Effect**	**Estimate**	**Std. Error**	**P value**	**95% CI**	
**Height**	0.0109834	0.0009582	<.001	0.0091053	0.0128615
**BMI$^{-3.6}$**	-3642.842	521.5231	<.001	-4665.009	-2620.675
**Sex male**	-0.4839596	0.155058	0.002	-0.7878677	-0.1800515
**Height male**	0.0038111	0.0009922	<.001	0.0018665	0.0057557
**Intercept**	2.581131	0.155561	<.001	2.276237	2.886025

Mixed-effects model for oxygen pulse

Table 9 Simple defects. Note: a dummy variable male has the value 1 if the individual is male, otherwise 0.

**Log(O_2 pulse)**					
**Effect**	**Estimate**	**Std. Error**	**P value**	**95% CI**	
**Height**	0.0146831	0.0004509	<.001	0.0137994	0.0155667
**$BMI^{-1.7}$**	-42.58742	3.69054	<.001	-49.82075	-35.3541
**Height male**	0.0009018	0.0000871	<.001	0.0007311	0.0010726
**Software Vyntus CPX**	-0.1793433	0.0254751	<.001	-0.2292735	-0.1294131
**Hospital Haukeland**	0.1046776	0.0135137	<.001	0.0781913	0.1311639
**Intercept**	0.2350272	0.0878036	0.007	0.0629354	0.4071191

Mixed-effects model for ventilatory efficiency

Table 10 Simple defects. Note: a dummy variable has the value 1 if the individual is male, otherwise 0.

**VE/VCO2 $slope^{-0.4}$**					
**Effect**	**Estimate**	**Std. Error**	**P value**	**95% CI**	
**Height**	0.0005124	0.0000839	<.001	0.000347	0.0006778
**Log(bmi)**	0.0170638	0.0042984	<.001	0.0086249	0.0255026
**Sex male**	0.0294384	0.0139676	0.036	0.0019002	0.0569766
**Height male**	-0.0001712	0.0000899	0.058	-0.0003486	6.21E-06
**Software Vyntus CPX**	-0.0143388	0.002699	<.001	-0.0196292	-0.0090485
**Hospital Haukeland**	0.0126451	0.001371	<.001	0.0099546	0.0153355
**Intercept**	0.1285197	0.0136275	<.001	0.1017916	0.1552479

Mixed-effects model for breathing frequency

Table 11 Simple defects. Note: a dummy variable male has the value 1 if the individual is male, otherwise 0.

**Breathing $frequency^{0.4}$**					
**Effect**	**Estimate**	**Std. Error**	**P value**	**95% CI**	
**Height**	-0.0114363	0.0007874	<.001	-0.0129796	-0.0098931
**Height male**	0.0007431	0.0002093	<.001	0.0003329	0.0011533
**Hospital Haukeland**	-0.1421088	0.0315442	<.001	-0.2039342	-0.0802833
**Intercept**	6.693345	0.1203646	<.001	6.457435	6.929256

Moderate defects

Weighted averages by hospital and software configurations

Table 12 Moderate defects. Weighted average for configurations of hospital and software.

Configuration	Hospital	Software	Weighting
1	Haukeland	Oxycon Pro/Sensor Medics Vmax	29.38 %
2	Haukeland	Vyntus CPX	5.63 %
3	Oslo	Oxycon Pro/Sensor Medics Vmax	64.39 %
4	Oslo	Vyntus CPX	0.60 %

Table 13 Moderate defects. Weighted average for hospital.

Configuration	Hospital	Weighting
1	Haukeland	35.01%
2	Oslo	64.99%

Table 14 Moderate defects. Weighted average for software.

Configuration	Software	Weighting
1	Oxycon Pro/Sensor Medics Vmax	93.77%
2	Vyntus CPX	6.23%

Intraclass correlation coefficients

Table 15 Intraclass correlation coefficients (ICC), variance of random intercept, and residual variance for each outcome in the mixed-effects models for moderate defects

Outcome	ICC	Variance of random intercept	Residual variance
**$\dot{V}$O2peak mL·min^{-1}**	0.587	0.0214062	0.0150855
**$\dot{V}$O2peak mL·kg^{-1}min^{-1}**	0.520	25.86781	23.88351
**Heart rate**	0.665	3.54e+14	1.79e+14
**Ventilation**	0.478	0.0190899	0.0208855
**Oxygen pulse**	0.496	0.0152225	0.0154626
**Ventilatory efficiency**	0.262	0.00006978	0.00019617
**Breathing frequency**	0.543	0.752893	0.6341427

Mixed-effects model for $\dot{V}O_2$peak (mL·min^{-1})

Table 16 Moderate defects. Note: a dummy variable male has the value 1 if the individual is male, otherwise 0.

**Log($\dot{V}O_2$ mL·min^{-1})**					
**Effect**	**Estimate**	**Std. Error**	**P value**	**95% CI**	
**Height**	0.0136715	0.0006271	<.001	0.0124425	0.0149005
**Log(BMI)**	0.3949761	0.0636892	<.001	0.2701476	0.5198046
**Height male**	0.0010347	0.0001348	<.001	0.0007705	0.0012989
**Software Vyntus CPX**	-0.0829085	0.032509	0.011	-0.1466249	-0.019192
**Hospital Haukeland**	0.088169	0.0220179	<.001	0.0450147	0.1313232
**Intercept**	4.069768	0.1396089	<.001	3.796139	4.343396

Mixed-effects model for $\dot{V}O_2peak$ ($mL \cdot kg^{-1} min^{-1}$)

Table 17 Moderate defects. Log-transformation on BMI. Note: a dummy variable male has the value 1 if the individual is male, otherwise 0.

**$\dot{V}O_2$ $mL \cdot kg^{-1} min^{-1}$**					
**Effect**	**Estimate**	**Std. Error**	**P value**	**95% CI**	
**Log(BMI)**	-21.99611	1.740452	<.001	-25.40733	-18.58489
**Height male**	0.0430605	0.0047544	<.001	0.0337421	0.0523789
**Software Vyntus CPX**	-3.504218	1.242076	0.005	-5.938642	-1.069794
**Hospital Haukeland**	3.483406	0.8113671	<.001	1.893156	5.073656
**Intercept**	99.9302	5.127618	<.001	89.88026	109.9802

Mixed-effects model for heart rate

Table 18 Moderate defects.

**Heart rate[5]**					
**Effect**	**Estimate**	**Std. Error**	**P value**	**95% CI**	
**Height**	9.90E+08	2.19E+08	<.001	5.60E+08	1.42E+09
**BMI**	-2.86E+09	1.15E+09	0.013	-5.10E+09	-6.13E+08
**Intercept**	1.40E+11	2.57E+10	<.001	8.95E+10	1.90E+11

Mixed-effects model for ventilation

Table 19 Moderate defects. Note: a dummy variable male has the value 1 if the individual is male, otherwise 0.

**Log(Ventilation)**					
**Effect**	**Estimate**	**Std. Error**	**P value**	**95% CI**	
**Height**	0.0118031	0.000937	<.001	0.0099667	0.0136396
**Log(BMI)**	0.3617417	0.0671844	<.001	0.2300626	0.4934208
**Sex male**	-0.3458141	0.1559535	0.027	-0.6514773	-0.040151
**Height male**	0.003166	0.0009997	0.002	0.0012067	0.0051254
**Intercept**	1.202455	0.1711635	<.001	0.8669809	1.537929

Mixed-effects model for oxygen pulse

Table 20 Moderate defects. Note: a dummy variable male has the value 1 if the individual is male, otherwise 0.

**Log(O_2 pulse)**					
**Effect**	**Estimate**	**Std. Error**	**P value**	**95% CI**	
**Height**	0.0125909	0.0005958	<.001	0.0114231	0.0137586
**Log(BMI)**	0.4460709	0.0591492	<.001	0.3301406	0.5620013
**Height male**	0.0010929	0.0001219	<.001	0.000854	0.0013318
**Software Vyntus CPX**	-0.0851175	0.0312816	0.007	-0.1464283	-0.0238066
**Hospital Haukeland**	0.0796701	0.020127	<.001	0.040222	0.1191182
**Intercept**	-1.143238	0.1312174	<.001	-1.400419	-0.8860566

Mixed-effects model for ventilatory efficiency

Table 21 Moderate defects.

VE/VCO2 slope$^{-0.4}$					
**Effect**	**Estimate**	**Std. Error**	**P value**	**95% CI**	
**Height**	0.0003922	0.000051	<.001	0.000291	0.0004934
**Software Vyntus CPX**	-0.0152721	0.0034107	<.001	-0.0219808	-0.0085634
**Hospital Haukeland**	0.0171314	0.0021677	<.001	0.012825	0.0214378
**Intercept**	0.1956142	0.0081168	<.001	0.1795195	0.2117088

Mixed-effects model for breathing frequency

Table 22 Moderate defects. Note: a dummy variable male has the value 1 if the individual is male, otherwise 0.

Breathing frequency$^{0.6}$					
**Effect**	**Estimate**	**Std. Error**	**P value**	**95% CI**	
**Height**	-0.037375	0.0045105	<.001	-0.0462154	-0.0285347
**Sex male**	-1.778892	0.8928496	0.046	-3.528845	-0.0289391
**Height male**	0.0134113	0.005712	0.019	0.0022161	0.0246066
**Hospital Haukeland**	-0.3806323	0.1326735	0.004	-0.6406676	-0.1205969
**Intercept**	16.65239	0.6998996	<.001	15.28061	18.02417

Univentricular defects with a Fontan circulation

Weighted averages by hospital and software configurations

Table 23 Fontan. Weighted average for configurations of hospital and software.

Configuration	Hospital	Software	Weighting
1	Haukeland	Oxycon Pro/Sensor Medics Vmax	16.97 %
2	Haukeland	Vyntus CPX	3.97 %
3	Oslo	Oxycon Pro/Sensor Medics Vmax	78.34 %
4	Oslo	Vyntus CPX	0.72 %

Table 24 Fontan. Weighted average for hospital.

Configuration	Hospital	Weighting
1	Haukeland	20.94%
2	Oslo	79.06%

Table 25 Fontan. Weighted average for software.

Configuration	Software	Weighting
1	Oxycon Pro/Sensor Medics Vmax	95.31%
2	Vyntus CPX	4.69%

Intraclass correlation coefficients

Table 26 Intraclass correlation coefficients (ICC), variance of random intercept, and residual variance for each outcome in the mixed-effects models for Fontan.

Outcome	ICC	Variance of random intercept	Residual variance
**V̇O2peak mL·min^{-1}**	0.659	0.0316358	0.016414
**V̇O2peak mL·kg^{-1}min^{-1}**	0.645	31.47055	17.35839
**Heart rate**	0.665	3.54e+14	1.79e+14
**Ventilation**	0.484	0.0227521	0.0243095
**Oxygen pulse**	0.660	0.0295089	0.0152227
**Ventilatory efficiency**	0.409	0.01045	0.01512
**Breathing frequency**	0.552	0.0185849	0.0150608

Mixed-effects model for V̇O_2peak (mL·min^{-1})

Table 27 Fontan circulation. Log-transformation for V̇O2peak and BMI. Note: a dummy variable male has the value 1 if the individual is male, otherwise 0.

**Log(V̇O_2 mL·min^{-1})**					
**Effect**	**Estimate**	**Std. Error**	**P value**	**95% CI**	
**Height**	0.0142453	0.0008327	<.001	0.0126132	0.0158773
**Log(BMI)**	0.3543394	0.1360816	0.009	0.0876243	0.6210545
**Sex male**	-0.8410274	0.4758941	0.077	-1.773763	0.0917078
**Log(BMI male)**	0.3348171	0.1626377	0.040	0.0160529	0.6535812
**Software Vyntus CPX**	-0.1212021	0.0612643	0.048	-0.241278	-0.0011263
**Hospital Haukeland**	0.0948334	0.0390418	0.015	0.0183128	0.1713539
**Intercept**	3.929859	0.3692452	<.001	3.206151	4.653566

Mixed-effects model for V̇O_2peak (mL·kg^{-1}min^{-1})

Table 28 Fontan circulation. Note: a dummy variable male has the value 1 if the individual is male, otherwise 0.

**V̇O_2 mL·kg^{-1}min^{-1}**					
**Effect**	**Estimate**	**Std. Error**	**P value**	**95% CI**	
**BMI**	-0.6682767	0.1350507	<.001	-0.9329713	-0.4035822
**Height male**	0.0329825	0.0067136	<.001	0.0198241	0.046141
**Software Vyntus CPX**	-4.52337	1.934238	0.019	-8.314407	-0.7323338
**Hospital Haukeland**	3.745683	1.243739	0.003	1.308	6.183366
**Intercept**	42.38803	2.678734	<.001	37.13781	47.63826

Mixed-effects model for heart rate

Table 29 Fontan circulation. Power-transformation of 3.5 for heart rate. Note: a dummy variable male has the value 1 if the individual is male, otherwise 0.

$\text{Heart rate}^{3.5}$					
**Effect**	**Estimate**	**Std. Error**	**P value**	**95% CI**	
**Height**	-144400.5	77584.03	0.063	-296462.4	7661.455
**Sex male**	-3.81E+07	1.29E+07	0.003	-6.35E+07	-1.27E+07
**BMI male**	2076971	682107.5	0.002	740064.6	3413877
**Software Vyntus CPX**	1.24E+07	6171610	0.045	286309.4	2.45E+07
**Intercept**	9.75E+07	1.20E+07	<.001	7.39E+07	1.21E+08

Mixed-effects model for ventilation

Table 30: Fontan. Log-transformation on ventilation and BMI. Note: a dummy variable male has the value 1 if the individual is male, otherwise 0.

**Log(Ventilation)**					
**Effect**	**Estimate**	**Std. Error**	**P value**	**95% CI**	
**Height**	0.0131873	0.0009373	<.001	0.0113503	0.0150243
**Log(BMI)**	0.3613543	0.1089674	0.001	0.1477822	0.5749265
**Height male**	0.0007153	0.0002184	0.001	0.0002873	0.0011434
**Intercept**	0.9744558	0.2539835	<.001	0.4766574	1.472254

Mixed-effects model for oxygen pulse

Table 31: Fontan. Log-transformation on O2-pulse. Note: a dummy variable male has the value 1 if the individual is male, otherwise 0.

**Log(O_2 pulse)**					
**Effect**	**Estimate**	**Std. Error**	**P value**	**95% CI**	
**Height**	0.0152299	0.0007706	<.001	0.0137195	0.0167403
**BMI**	0.0158716	0.0051144	0.002	0.0058476	0.0258957
**BMI male**	0.0071081	0.0017693	<.001	0.0036404	0.0105759
**Software Vyntus CPX**	-0.1669066	0.0588834	0.005	-0.2823159	-0.0514973
**Hospital Haukeland**	0.0745283	0.0375552	0.047	0.0009214	0.1481352
**Intercept**	-0.6453765	0.0981614	<.001	-0.8377694	-0.4529836

Mixed-effects model for ventilatory efficiency

Table 32 Fontan. Log transformation on ventilatory efficiency. Note: a dummy variable male has the value 1 if the individual is male, otherwise 0.

**Log(VE/VCO2 slope)**					
**Effect**	**Estimate**	**Std. Error**	**P value**	**95% CI**	
**Height**	-0.0058539	0.0011404	<.001	-0.0080912	-0.0036167
**Sex male**	-0.6899319	0.202	0.001	-1.08637	-0.2934941
**Height male**	0.0041379	0.0013105	0.002	0.0015664	0.0067095
**Software Vyntus CPX**	0.1335418	0.0541759	0.014	0.0269421	0.2401415
**Hospital Haukeland**	-0.1643959	0.0343414	<.001	-0.2323451	-0.0964467
**Intercept**	4.505025	0.1739446	<.001	4.163772	4.846278

Mixed-effects model for breathing frequency

Table 33 Fontan circulation. Log-transformation on breathing frequency and BMI. Note: a dummy variable male has the value 1 if the individual is male, otherwise 0.

**Log(Breathing frequency)**					
**Effect**	**Estimate**	**Std. Error**	**P value**	**95% CI**	
**Height**	-0.0044619	0.0005702	<.001	-0.0055795	-0.0033443
**Log(BMI male)**	0.0225936	0.0097797	0.021	0.0034258	0.417615
**Hospital Haukeland**	-0.0820773	0.0326546	0.012	-0.1460791	-0.0180756
**Intercept**	4.609728	0.0893711	<.001	4.434564	4.784892
