# Supplementary material for: Reference models for individualized assessment of cardiorespiratory fitness in children and adolescents with congenital heart disease: a retrospective multicentre study
Source: Eur J Pediatr. 2025 Jun 26;184(7):450. doi: 10.1007/s00431-025-06270-x (PMC12202686; doi:10.1007/s00431-025-06270-x)
Supplement: Supplementary file 4 — (PDF 470 kb) [file 431_2025_6270_MOESM4_ESM.pdf]

Online supplement 4

Reference models for individualized assessment of cardiorespiratory fitness in children and adolescents with congenital heart disease: a retrospective multicentre study

European Journal of Pediatrics

Vibeke Klungerbo[a,b], Asle Hirth[c], Per Morten Fredriksen[d,e], René Holst[f], Elisabeth Edvardsen[g], Henrik Holmstrøm[b], Thomas Möller[a]

a) Department of Paediatric Cardiology, Oslo University Hospital, Oslo, Norway
b) Institute of Clinical Medicine, Faculty of Medicine, University of Oslo, Oslo, Norway
c) Department of Paediatrics, Haukeland University Hospital, Bergen, Norway
d) Faculty of Applied Ecology, Agricultural Sciences and Biotechnology, University of Inland Norway, Hamar, Norway
e) Faculty of Health, Welfare and Organization, Østfold University College, Fredrikstad, Norway
f) Department of Biostatistics, Institute of Basic Medical Sciences, University of Oslo, Oslo, Norway
g) Department of Pulmonary Medicine, Oslo University Hospital, Oslo, Norway

<u>Corresponding author:</u>

Vibeke Klungerbo

Dept. of Paediatric Cardiology

Oslo University Hospital

P.O. Box 4950 Nydalen, 0424 Oslo, Norway

Phone: +47 23070000

Fax: +47 23072330

E-mail: vibklu@ous-hf.no

ORCID: 0000-0003-0980-0971

Supplement 4

Simple defects

Covariance matrix for V̇O$_2$peak (mL·min^{-1})

Table 1 Covariance matrix for V̇O2peak in mL·min^{-1} for Simple defects.

	Log(V̇O$_2$peak mL·min^{-1}) : height	Log(V̇O$_2$peak mL·min^{-1}) : log(bmi)	Log(V̇O$_2$peak mL·min^{-1}) : height male	Log(V̇O$_2$peak mL·min^{-1}) : software Vyntus CPX	Log(V̇O$_2$peak mL·min^{-1}) : hospital Haukeland	Log(V̇O$_2$peak mL·min^{-1}) : _cons
**Log(V̇O$_2$peak mL·min^{-1}) : height**	2.11E-07	-0.00001263	-1.46E-08	-9.02E-07	-1.49E-07	5.55E-06
**Log(V̇O$_2$peak mL·min^{-1}) : log(bmi)**	-0.00001263	0.0018624	6.41E+07	-0.00001219	0.00004482	-0.00358706
**Log(V̇O$_2$peak mL·min^{-1}) : height male**	-1.46E-08	6.41E-07	8.52E-09	1.56E-07	-1.58E-08	-4.38E-07
**Log(V̇O$_2$peak mL·min^{-1}) : software Vyntus CPX**	-9.02E-07	-0.00001219	1.56E-07	7.23E-04	-9.26E-05	1.58E-04
**Log(V̇O$_2$peak mL·min^{-1}) : hospital Haukeland**	-1.49E-07	0.00004482	-1.58E-08	-9.26E-05	2.05E-04	-1.84E-04
**Log(V̇O$_2$peak mL·min^{-1}) : _cons**	5.55E-06	-0.00358706	-4.38E-07	1.58E-04	-1.84E-04	0.00986512

Covariance matrix for V̇O$_2$peak (mL·kg^{-1}min^{-1})

Table 2 Covariance matrix for V̇O2peak in mL·kg^{-1}min^{-1} for Simple defects.

	V̇O$_2$peak mL·kg^{-}1min^{-1} : height	V̇O$_2$peak mL·kg^{-}1min^{-1} : bmi	V̇O$_2$peak mL·kg^{-}1min^{-1} : height male	V̇O$_2$peak mL·kg^{-}1min^{-1} : software Vyntus CPX	V̇O$_2$peak mL·kg^{-}1min^{-1} : hospital Haukeland	V̇O$_2$peak mL·kg^{-}1min^{-1} : _cons
**V̇O$_2$peak mL·kg^{-}1min^{-1} : height**	0.00035024	-0.00098016	-0.00002489	-0.00171601	-0.00015618	-0.03344335
**V̇O$_2$peak mL·kg^{-}1min^{-1} : bmi**	-0.00098016	0.00757501	0.0000504	-0.00036311	0.00335704	0.0006148
**V̇O$_2$peak mL·kg^{-}1min^{-1} : height male**	-0.00002489	0.0000504	0.00001519	0.00028271	-0.00003328	0.00142169
**V̇O$_2$peak mL·kg^{-}1min^{-1} : software Vyntus CPX**	-0.00171601	-0.00036311	0.00028271	1.2854802	-0.1644319	0.24078036
**V̇O$_2$peak mL·kg^{-}1min^{-1} : hospital Haukeland**	-0.00015618	0.00335704	-0.00003328	-0.1644319	0.36629488	-0.17410341
**V̇O$_2$peak mL·kg^{-}1min^{-1} : _cons**	-0.03344335	0.0006148	0.00142169	0.24078036	-0.17410341	5.2525623

Covariance matrix for heart rate

Table 3 Covariance matrix for heart rate for Simple defects

	**Heart rate$^{4.3}$: height**	**Heart rate$^{4.3}$: _cons**
**Heart rate$^{4.3}$: height**	1.07E+13	-1.69E+15
**Heart rate$^{4.3}$: _cons**	-1.69E+15	2.70E+17

Covariance matrix for ventilation

Table 4 Covariance matrix for ventilation for Simple defects

	**Log(Ventilation) : height**	**Log(Ventilation) : BMI$^{-3.6}$**	**Log(Ventilation) : sex male**	**Log(Ventilation) : height male**	**Log(Ventilation) : _cons**
**Log(Ventilation) : height**	9.18E-07	0.21897971	0.00011749	-7.69E-07	-0.00014793
**Log(Ventilation) : BMI$^{-3.6}$**	0.21897971	271986.39	3.8461203	-0.03392181	-41.647763
**Log(Ventilation) : sex male**	0.00011749	3.8461203	0.02404299	-0.00015287	-0.01841087
**Log(Ventilation) : height male**	-7.69E-07	-0.03392181	-0.00015287	9.84E-07	0.00011959
**Log(Ventilation) : _cons**	-0.00014793	-41.647763	-0.01841087	0.00011959	0.02419922

Covariance matrix for oxygen pulse

Table 5 Covariance matrix for oxygen pulse for Simple defects

	**Log(O_2 pulse) : height**	**Log(O_2 pulse) : BMI$^{-1.7}$**	**Log(O_2 pulse) : height male**	**Log(O_2 pulse) : software Vyntus CPX**	**Log(O_2 pulse) : hospital Haukeland**	**Log(O_2 pulse) : _cons**
**Log(O_2 pulse) : height**	2.03E-07	0.00110813	-1.36E-08	-7.20E-07	-2.09E-07	-3.82E-05
**Log(O_2 pulse) : BMI$^{-1.7}$**	0.00110813	13.620085	-0.00005427	0.0019093	-0.00427112	-0.26224647
**Log(O_2 pulse) : height male**	-1.36E-08	-0.00005427	7.59E-09	1.36E-07	-1.10E-08	1.76E-06
**Log(O_2 pulse) : software Vyntus CPX**	-7.20E-07	0.0019093	1.36E-07	0.00064898	-0.00008335	0.00008247
**Log(O_2 pulse) : hospital Haukeland**	-2.09E-07	-0.00427112	-1.10E-08	-0.00008335	1.83E-04	-4.39E-06
**Log(O_2 pulse) : _cons**	-3.82E-05	-0.26224647	1.76E-06	0.00008247	-4.39E-06	0.00770947

Covariance matrix for breathing frequency

Table 6 Covariance matrix for breathing frequency for Simple defects

	**Breathing frequency$^{0.4}$: height**	**Breathing frequency$^{0.4}$: height male**	**Breathing frequency$^{0.4}$: hospital Haukeland**	**Breathing frequency$^{0.4}$: _cons**
**Breathing frequency$^{0.4}$: height**	6.20E-07	-5.14E-08	-4.42E-07	-9.21E-05
**Breathing frequency$^{0.4}$: height male**	-5.14E-08	4.38E-08	5.01E-08	3.66E-06
**Breathing frequency$^{0.4}$: hospital Haukeland**	-4.42E-07	5.01E-08	0.00099503	-0.00035021
**Breathing frequency$^{0.4}$: _cons**	-9.21E-05	3.66E-06	-0.00035021	0.01448764

Moderate defects

Covariance matrix for V̇O₂peak (mL·min^{-1})

Table 7 Covariance matrix for V̇O2peak in mL·min^{-1} for Moderate defects

	**Log(V̇O_2peak mL·min^{-1}) : height**	**Log(V̇O_2peak mL·min^{-1}) : log(bmi)**	**Log(V̇O_2peak mL·min^{-1}) : height male**	**Log(V̇O_2peak mL·min^{-1}) : software Vyntus CPX**	**Log(V̇O_2peak mL·min^{-1}) : hospital Haukeland**	**Log(V̇O_2peak mL·min^{-1}) : _cons**
**Log(V̇O_2peak mL·min^{-1}) : height**	3.93E-07	-0.00002769	-2.20E-08	-1.67E-06	3.41E-07	2.19E-05
**Log(V̇O_2peak mL·min^{-1}) : log(bmi)**	-0.00002769	0.00405631	7.83E-07	-0.00004597	0.00017076	-0.00769375
**Log(V̇O_2peak mL·min^{-1}) : height male**	-2.20E-08	7.83E-07	1.82E-08	-1.90E-08	9.34E-08	-5.73E-07
**Log(V̇O_2peak mL·min^{-1}) : software Vyntus CPX**	-1.67E-06	-0.00004597	-1.90E-08	0.00105683	-0.00018295	0.00038466
**Log(V̇O_2peak mL·min^{-1}) : hospital Haukeland**	3.41E-07	0.00017076	9.34E-08	-0.00018295	4.85E-04	-7.09E-04
**Log(V̇O_2peak mL·min^{-1}) : _cons**	2.19E-05	-0.00769375	-5.73E-07	0.00038466	-7.09E-04	0.01949065

Covariance matrix for $\dot{V}O_2$peak ($mL \cdot kg^{-1} min^{-1}$)

Table 8 Covariance matrix for $\dot{V}O2$peak in $mL \cdot kg^{-1} min^{-1}$ for Moderate defects

	$\dot{V}O_2$peak $mL \cdot kg^{-}1min^{-1}$: log(bmi)	$\dot{V}O_2$peak $mL \cdot kg^{-}1min^{-1}$: height male	$\dot{V}O_2$peak $mL \cdot kg^{-}1min^{-1}$: software Vyntus CPX	$\dot{V}O_2$peak $mL \cdot kg^{-}1min^{-1}$: hospital Haukeland	$\dot{V}O_2$peak $mL \cdot kg^{-}1min^{-1}$: _cons
**$\dot{V}O_2$peak $mL \cdot kg^{-}1min^{-1}$: log(bmi)**	3.0291717	-0.00101912	-0.21071336	0.27370221	-8.8546093
**$\dot{V}O_2$peak $mL \cdot kg^{-}1min^{-1}$: height male**	-0.00101912	0.0000226	-0.00015802	0.0001536	0.00085928
**$\dot{V}O_2$peak $mL \cdot kg^{-}1min^{-1}$: software Vyntus CPX**	-0.21071336	-0.00015802	1.5427521	-0.26612529	0.61523721
**$\dot{V}O_2$peak $mL \cdot kg^{-}1min^{-1}$: hospital Haukeland**	0.27370221	0.0001536	-0.26612529	0.65831658	-1.0159748
**$\dot{V}O_2$peak $mL \cdot kg^{-}1min^{-1}$: _cons**	-8.8546093	0.00085928	0.61523721	-1.0159748	26.29247

Covariance matrix for heart rate

Table 9 Covariance matrix for heart rate for Moderate defects

	Heart rate^5 : height	Heart rate^5 : bmi	Heart rate^5 : _cons
**Heart rate^5 : height**	4.80E+16	-1.68E+17	-4.28E+18
**Heart rate^5 : bmi**	-1.68E+17	1.31E+18	1.14E+18
**Heart rate^5 : _cons**	-4.28E+18	1.14E+18	6.59E+20

Covariance matrix for ventilation

Table 10 Covariance matrix for ventilation for Moderate defects

	Log(Ventilation) : height	Log(Ventilation) : log(bmi)	Log(Ventilation) : sex male	Log(Ventilation) : height male	Log(Ventilation) : _cons
**Log(Ventilation) : height**	8.78E-07	-0.00003363	0.00010017	-6.61E-07	-0.00003577
**Log(Ventilation) : log(bmi)**	-0.00003363	0.00451375	-0.00057525	4.48E-06	-0.00806025
**Log(Ventilation) : sex male**	0.00010017	-0.00057525	0.02432148	-0.0001544	-0.01388307
**Log(Ventilation) : height male**	-6.61E-07	4.48E-06	-0.0001544	9.99E-07	0.00008785
**Log(Ventilation) : _cons**	-0.00003577	-0.00806025	-0.01388307	0.00008785	0.02929694

Covariance matrix for oxygen pulse

Table 11 Covariance matrix for oxygen pulse for Moderate defects

	**Log(O_2 pulse) : height**	**Log(O_2 pulse) : log(bmi)**	**Log(O_2 pulse) : height male**	**Log(O_2 pulse) : software Vyntus CPX**	**Log(O_2 pulse) : hospital Haukeland**	**Log(O_2 pulse) : _cons**
**Log(O_2 pulse) : height**	3.55E-07	-0.00002384	-1.92E-08	-1.37E-06	3.54E-07	1.63E-05
**Log(O_2 pulse) : log(bmi)**	-0.00002384	0.00349863	6.64E-07	-0.00003482	0.00014633	-0.00664163
**Log(O_2 pulse) : height male**	-1.92E-08	6.64E-07	1.49E-08	-2.44E-08	7.54E-08	-3.54E-07
**Log(O_2 pulse) : software Vyntus CPX**	-1.37E-06	-0.00003482	-2.44E-08	0.00097854	-0.00016897	0.0003073
**Log(O_2 pulse) : hospital Haukeland**	3.54E-07	0.00014633	7.54E-08	-0.00016897	4.05E-04	-6.14E-04
**Log(O_2 pulse) : _cons**	1.63E-05	-0.00664163	-3.54E-07	0.0003073	-6.14E-04	0.01721801

Covariance matrix for breathing frequency

Table 12 Covariance matrix for breathing frequency for Moderate defects

	**Breathing frequency$^{0.6}$: height**	**Breathing frequency$^{0.6}$: sex male**	**Breathing frequency$^{0.6}$: height male**	**Breathing frequency$^{0.6}$: hospital Haukeland**	**Breathing frequency$^{0.6}$: _cons**
**Breathing frequency$^{0.6}$: height**	0.00002034	0.00308449	-0.00002022	0.00004208	-0.0031175
**Breathing frequency$^{0.6}$: sex male**	0.00308449	0.79718032	-0.00504493	-0.00113243	-0.47991971
**Breathing frequency$^{0.6}$: height male**	-0.00002022	-0.00504493	0.00003263	0.00001023	0.00307983
**Breathing frequency$^{0.6}$: hospital Haukeland**	0.00004208	-0.00113243	0.00001023	0.01760227	-0.0126732
**Breathing frequency$^{0.6}$: _cons**	-0.0031175	-0.47991971	0.00307983	-0.0126732	0.48985943

Univentricular defects with a Fontan circulation

Covariance matrix for V̇O_2peak (mL·min^{-1})

Table 13 Covariance matrix for V̇O2peak in mL·min^{-1} for univentricular defects with a Fontan circulation.

	**Log(V̇O_2peak mL·min^{-1}) : height**	**Log(V̇O_2peak mL·min^{-1}) : log(bmi)**	**Log(V̇O_2peak mL·min^{-1}) : sex male**	**Log(V̇O_2peak mL·min^{-1}) : log(bmi male)**	**Log(V̇O_2peak mL·min^{-1}) : software Vyntus CPX**	**Log(V̇O_2peak mL·min^{-1}) : hospital Haukeland**	**Log(V̇O_2peak mL·min^{-1}) : _cons**
**Log(V̇O_2peak mL·min^{-1}) : height**	6.93E-07	-0.00004553	0.0000618	-0.00002303	-8.39E-06	2.53E-06	0.00002892
**Log(V̇O_2peak mL·min^{-1}) : log(bmi)**	-0.00004553	0.01851821	0.04064398	-0.01371626	0.00094428	0.00035114	-0.0475786
**Log(V̇O_2peak mL·min^{-1}) : sex male**	0.0000618	0.04064398	0.22647517	-0.07718575	0.00191534	-0.00098601	-0.12919257
**Log(V̇O_2peak mL·min^{-1}) : log(bmi male)**	-0.00002303	-0.01371626	-0.07718575	0.02645103	-0.00059656	0.00034461	0.04368487
**Log(V̇O_2peak mL·min^{-1}) : software Vyntus CPX**	-8.39E-06	0.00094428	0.00191534	-0.00059656	0.00375332	-0.00058942	-0.00165901
**Log(V̇O_2peak mL·min^{-1}) : hospital Haukeland**	2.53E-06	0.00035114	-0.00098601	0.00034461	-0.00058942	0.00152426	-0.00167278
**Log(V̇O_2peak mL·min^{-1}) : _cons**	0.00002892	-0.0475786	-0.12919257	0.04368487	-0.00165901	-0.00167278	0.13634202

Covariance matrix for V̇O_2peak (mL·kg^{-1}min^{-1})

Table 14 Covariance matrix for V̇O2peak in mL·kg^{-1}min^{-1} for univentricular defects with a Fontan circulation.

	**V̇O_2peak mL·kg^{-}1min^{-1} : bmi**	**V̇O_2peak mL·kg^{-}1min^{-1} : height male**	**V̇O_2peak mL·kg^{-}1min^{-1} : software Vyntus CPX**	**V̇O_2peak mL·kg^{-}1min^{-1} : hospital Haukeland**	**V̇O_2peak mL·kg^{-}1min^{-1} : _cons**
**V̇O_2peak mL·kg^{-}1min^{-1} : bmi**	0.0182387	-0.00007529	-0.00583977	0.0383486	-0.34244845
**V̇O_2peak mL·kg^{-}1min^{-1} : height male**	-0.00007529	0.00004507	-0.00002117	0.00053872	-0.00285108
**V̇O_2peak mL·kg^{-}1min^{-1} : software Vyntus CPX**	-0.00583977	-0.00002117	3.741276	-0.57201401	0.0161844
**V̇O_2peak mL·kg^{-}1min^{-1} : hospital Haukeland**	0.0383486	0.00053872	-0.57201401	1.5468858	-1.0226426
**V̇O_2peak mL·kg^{-}1min^{-1} : _cons**	-0.34244845	-0.00285108	0.0161844	-1.0226426	7.1756135

Covariance matrix for heart rate

Table 15 Covariance matrix for heart rate for univentricular defects with a Fontan circulation

	**Heart rate$^{3.5}$: height**	**Heart rate$^{3.5}$: sex male**	**Heart rate$^{3.5}$: bmi male**	**Heart rate$^{3.5}$: software Vyntus CPX**	**Heart rate$^{3.5}$: _cons**
**Heart rate$^{3.5}$: height**	6.02E+09	5.37E+11	-3.09E+10	-5.22E+10	-9.05E+11
**Heart rate$^{3.5}$: sex male**	5.37E+11	1.68E+14	-8.47E+12	-2.27E+12	-8.87E+13
**Heart rate$^{3.5}$: bmi male**	-3.09E+10	-8.47E+12	4.65E+11	2.07E+11	4.65E+12
**Heart rate$^{3.5}$: software Vyntus CPX**	-5.22E+10	-2.27E+12	2.07E+11	3.81E+13	5.10E+12
**Heart rate$^{3.5}$: _cons**	-9.05E+11	-8.87E+13	4.65E+12	5.10E+12	1.44E+14

Covariance matrix for ventilation

Table 16 Covariance matrix for ventilation for univentricular defects with a Fontan circulation

	**Log(Ventilation) : height**	**Log(Ventilation) : log(bmi)**	**Log(Ventilation) : height male**	**Log(Ventilation) : _cons**
**Log(Ventilation) : height**	8.79E-07	-0.00006495	-7.64E-08	0.00006114
**Log(Ventilation) : log(bmi)**	-0.00006495	0.0118739	4.46E-06	-0.02505763
**Log(Ventilation) : height male**	-7.64E-08	4.46E-06	4.77E-08	-5.65E-06
**Log(Ventilation) : _cons**	0.00006114	-0.02505763	-5.65E-06	0.0645076

Covariance matrix for oxygen pulse

Table 17 Covariance matrix for oxygen pulse for univentricular defects with a Fontan circulation

	**Log(O_2 pulse) : height**	**Log(O_2 pulse) : bmi**	**Log(O_2 pulse) : bmi male**	**Log(O_2 pulse) : software Vyntus CPX**	**Log(O_2 pulse) : hospital Haukeland**	**Log(O_2 pulse) : _cons**
**Log(O_2 pulse) : height**	5.94E-07	-2.35E-06	-2.91E-07	-8.00E-06	2.91E-06	-4.45E-05
**Log(O_2 pulse) : bmi**	-2.35E-06	0.00002616	-1.88E-07	0.00002484	0.00002244	-0.00013148
**Log(O_2 pulse) : bmi male**	-2.91E-07	-1.88E-07	3.13E-06	6.95E-06	1.48E-06	1.35E-05
**Log(O_2 pulse) : software Vyntus CPX**	-8.00E-06	0.00002484	6.95E-06	0.00346725	-0.00054121	0.00060656
**Log(O_2 pulse) : hospital Haukeland**	2.91E-06	0.00002244	1.48E-06	-0.00054121	1.41E-03	-1.12E-03
**Log(O_2 pulse) : _cons**	-4.45E-05	-0.00013148	1.35E-05	0.00060656	-1.12E-03	0.00963567

Covariance matrix for breathing frequency

Table 18 Covariance matrix for breathing frequency for univentricular defects with a Fontan circulation

	**Log(Breathing frequency) : height**	**Log(Breathing frequency) : log(bmi male)**	**Log(Breathing frequency) : hospital Haukeland**	**Log(Breathing frequency) : _cons**
**Log(Breathing frequency) : height**	3.25E-07	-9.17E-07	3.64E-06	-4.93E-05
**Log(Breathing frequency) : log(bmi male)**	-9.17E-07	0.00009564	7.00E-06	-0.0000241
**Log(Breathing frequency) : hospital Haukeland**	3.64E-06	7.00E-06	0.00106632	-0.00077028
**Log(Breathing frequency) : _cons**	-4.93E-05	-0.0000241	-0.00077028	0.00798718
