# Supplementary material for: Reference models for individualized assessment of cardiorespiratory fitness in children and adolescents with congenital heart disease: a retrospective multicentre study
Source: Eur J Pediatr. 2025 Jun 26;184(7):450. doi: 10.1007/s00431-025-06270-x (PMC12202686; doi:10.1007/s00431-025-06270-x)
Supplement: Supplementary file 5 — (PDF 273 kb) [file 431_2025_6270_MOESM5_ESM.pdf]

Online supplement

Reference models for individualized assessment of cardiorespiratory fitness in children and adolescents with congenital heart disease: a retrospective multicentre study

European Journal of Pediatrics

Vibeke Klungerbo[a,b], Asle Hirth[c], Per Morten Fredriksen[d,e], René Holst[f], Elisabeth Edvardsen[g], Henrik Holmstrøm[b], Thomas Möller[a]

a) Department of Paediatric Cardiology, Oslo University Hospital, Oslo, Norway
b) Institute of Clinical Medicine, Faculty of Medicine, University of Oslo, Oslo, Norway
c) Department of Paediatrics, Haukeland University Hospital, Bergen, Norway
d) Faculty of Applied Ecology, Agricultural Sciences and Biotechnology, University of Inland Norway, Hamar, Norway
e) Faculty of Health, Welfare and Organization, Østfold University College, Fredrikstad, Norway
f) Department of Biostatistics, Institute of Basic Medical Sciences, University of Oslo, Oslo, Norway
g) Department of Pulmonary Medicine, Oslo University Hospital, Oslo, Norway

Corresponding author:

Vibeke Klungerbo

Dept. of Paediatric Cardiology

Oslo University Hospital

P.O. Box 4950 Nydalen, 0424 Oslo, Norway

Phone: +47 23070000

Fax: +47 23072330

E-mail: vibklu@ous-hf.no

ORCID: 0000-0003-0980-0971

Supplement 5

<u>Calculations for the example patient in the results-section:</u>

Configuration 1:

$42.38803 + (-0.6682767 * 21) + (0.0329825 * 160) + (-4.52337 * 0) + (3.745683 * 1) = 37.38\ \text{mL·kg}^{-1}\text{·min}^{-1}$

Configuration 2:

$42.38803 + (-0.6682767 * 21) + (0.0329825 * 160) + (-4.52337 * 1) + (3.745683 * 1) = 32.85\ \text{mL·kg}^{-1}\text{· min}^{-1}$

Configuration 3:

$42.38803 + (-0.6682767 * 21) + (0.0329825 * 160) + (-4.52337 * 0) + (3.745683 * 0) = 33.63\ \text{mL·kg}^{-1}\text{· min}^{-1}$

Configuration 4:

$42.38803 + (-0.6682767 * 21) + (0.0329825 * 160) + (-4.52337 * 1) + (3.745683 * 0) = 29.11\ \text{mL·kg}^{-1}\text{· min}^{-1}$

Table 1. Weighted average for configurations of hospital and software for Fontans.

Configuration	Hospital	Software	Weighting w_i	Prediction $\hat{Y}_i$
1	Haukeland	Oxycon Pro/ Sensor Medics Vmax	16.97 %	37.38
2	Haukeland	Vyntus CPX	3.97 %	32.85
3	Oslo	Oxycon Pro/ Sensor Medics Vmax	78.34 %	33.63
4	Oslo	Vyntus CPX	0.72 %	29.11

$$\hat{Y} = w_1 \cdot \hat{Y}_1 + w_2 \cdot \hat{Y}_2 + w_3 \cdot \hat{Y}_3 + w_4 \cdot \hat{Y}_4$$

Whereas $\hat{Y}$ represents $\dot{V}O_2$peak, w_i is the weighting and $\hat{Y}_i$ is the prediction for the *j*'th configuration.

$(0.1697 \cdot 37.38 + 0.0397 \cdot 32.85 + 0.7834 \cdot 33.63 + 0.0072 \cdot 29.11) = 34.20\ \text{mL·kg}^{-1}\text{·min}^{-1}$
