# Supplementary material for: Reference models for individualized assessment of cardiorespiratory fitness in children and adolescents with congenital heart disease: a retrospective multicentre study
Source: Eur J Pediatr. 2025 Jun 26;184(7):450. doi: 10.1007/s00431-025-06270-x (PMC12202686; doi:10.1007/s00431-025-06270-x)
Supplement: Supplementary file 6 — (PDF 230 kb) [file 431_2025_6270_MOESM6_ESM.pdf]

Online supplement 6

Reference models for individualized assessment of cardiorespiratory fitness in children and adolescents with congenital heart disease: a retrospective multicentre study

European Journal of Pediatrics

Vibeke Klungerbo[a,b], Asle Hirth[c], Per Morten Fredriksen[d,e], René Holst[f], Elisabeth Edvardsen[g], Henrik Holmstrøm[b], Thomas Möller[a]

a) Department of Paediatric Cardiology, Oslo University Hospital, Oslo, Norway
b) Institute of Clinical Medicine, Faculty of Medicine, University of Oslo, Oslo, Norway
c) Department of Paediatrics, Haukeland University Hospital, Bergen, Norway
d) Faculty of Applied Ecology, Agricultural Sciences and Biotechnology, University of Inland Norway, Hamar, Norway
e) Faculty of Health, Welfare and Organization, Østfold University College, Fredrikstad, Norway
f) Department of Biostatistics, Institute of Basic Medical Sciences, University of Oslo, Oslo, Norway
g) Department of Pulmonary Medicine, Oslo University Hospital, Oslo, Norway

Corresponding author:

Vibeke Klungerbo

Dept. of Paediatric Cardiology

Oslo University Hospital

P.O. Box 4950 Nydalen, 0424 Oslo, Norway

Phone: +47 23070000

Fax: +47 23072330

E-mail: vibklu@ous-hf.no

ORCID: 0000-0003-0980-0971

Web-based app - Calculator

URL: ocbe.shinyapps.io/kids-chd/

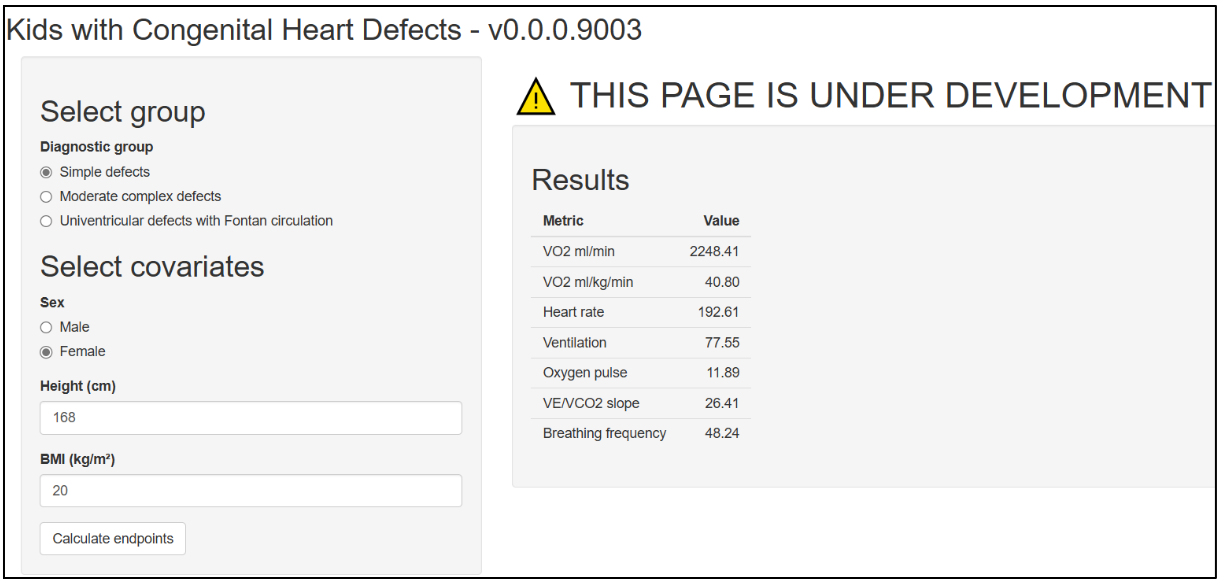

Figure 1 Screenshot of the web-based calculator 16.05.2025.

Description of the interface

The web-based calculator titled "Kids with Congenital Heart Defects" is designed to estimate endpoints from the cardiopulmonary exercise test based on patient characteristics, providing individualized reference values. On the left side of the interface, users can select a diagnostic group (Simple defects, Moderate complex defects, or Univentricular defects with Fontan circulation), enter covariates such as sex, height (cm), and BMI (kg/m²), and then click *"Calculate endpoints".* On the right, the *Results* panel displays individualized reference values for $\dot{V}O_2$peak (mL·min^{-1} and mL·kg^{-1}min^{-1}), heart rate, ventilation, oxygen pulse, ventilatory efficiency (V_E/VCO_2 slope), and breathing frequency. As indicated by the warning, the page is still under development.
